# Preparation of Amino-Functionalized Mesoporous SBA-15 Nanoparticles and the Improved Adsorption of Tannic Acid in Wastewater

**DOI:** 10.3390/nano12050791

**Published:** 2022-02-26

**Authors:** Tzong-Horng Liou, Guan-Wei Chen, Shang Yang

**Affiliations:** 1 Department of Chemical Engineering, Ming Chi University of Technology, 84 Gungjuan Road, Taishan District, New Taipei City 24301, Taiwan; u06137029@gmail.com (G.-W.C.); a0981578295@gmail.com (S.Y.); 2 Battery Research Center of Green Energy, Ming Chi University of Technology, 84 Gungjuan Road, Taishan District, New Taipei City 24301, Taiwan

**Keywords:** SBA-15, amino groups, adsorption, tannic acid, mesostructure

## Abstract

Ordered mesoporous Santa Barbara amorphous (SBA-15) materials have high surface areas and are widely used in adsorption, separation, filtration, and heterogeneous catalytic processes. However, SBA-15 surfaces contain hydroxyl groups that are unsuited to the adsorption of organic pollutants; thus, SBA-15 must be chemically modified to promote its adsorption activity. In this study, amino-functionalized nanoporous SBA-15 was fabricated by employing sodium silicate as a precursor. The structural characteristics of the prepared composites were examined using thermogravimetric analysis, X-ray diffraction, Fourier transform infrared spectrometry, field-emission scanning electron microscopy, transmission electron microscopy, and surface area analysis. The prepared SBA-15 had a large pore size (6.46–7.60 nm), large pore volume (1.037–1.105 cm^3^/g), and high surface area (546–766 m^2^/g). Functionalization caused a reduction in the SBA-15 pore volume and surface area, whereas amino groups that promoted an interaction between adsorbates and solids facilitated solute adsorption. The adsorption of tannic acid (TA) onto amino-modified silica composites (SBA-15 and 3-aminopropyltriethoxysilane (SBA-15/APTES) and SBA-15 and pentaethylenehexamine (SBA-15/PEHA)) was studied. Their adsorption capacities were affected by solution temperature, solution pH, agitation speed, adsorbent dosage, and initial TA concentration. The maximum adsorption capacities for SBA-15/APTES and SBA-15/PEHA were 485.18 and 413.33 mg/g, respectively, with SBA-15/APTES exhibiting ultrafast removal of TA (98.61% removal rate at 15 min). In addition, this study explored the thermodynamics, adsorption isotherms, and kinetics. A comparison of two types of amino-functionalized SBA-15 was used for the first time to adsorb TA, which providing valuable information on TA adsorption on high adsorption capacity materials in water media.

## 1. Introduction

Nanomaterials are widely applied in many fields such as pharmaceutics, energy, semiconductors, and the environment [1,2]. The synthesis of SBA-15 nanomaterials has attracted considerable attention since Chmelka et al. developed the process in 1998 [3]. According to the International Union of Pure and Applied Chemistry, mesoporous materials have pore sizes of 2–50 nm, and SBA-15 materials possess uniform mesopores, a high pore volume, large specific surface area, and stability, making them suitable for numerous applications related to adsorption [4], catalysis [5], tissue engineering [6], drug release [7], CO_2_ capture [8], and energy cells [9]. SBA-15 is prepared in an acidic environment through the interaction between a surfactant template and silicon precursor, producing a two-dimensional and well-ordered hexagonal structure. As compared with microporous materials, such as zeolites, mesoporous SBA-15 material has large pore sizes (approximately 10–30 nm), which can decrease mass transfer resistance for large adsorbates, making it a potential material for the adsorption of wastewater. Sodium silicate (Na_2_SiO_3_) and tetraethyl orthosilicate (TEOS) are both typically used as a silicon source to synthesize SBA-15 materials [10,11]. However, the high-priced TEOS reagent increases the cost of raw materials. By contrast, sodium silicate is a low-cost silicon source for the mass manufacture of SBA-15. Converting sodium silicate into mesoporous silica materials can eliminate hazardous pollutants from wastewater and air.

Tannic acid (TA) is an organic compound that is frequently found in surface and drinking water [12]. TA is also a raw material used in many industries, including in the manufacturing of ink, medicines, paper, and leather products [13]. Recently, TA has raised concerns because of its toxicity to aquatic animals and plants [14]. Furthermore, in drinking water, TA can interact with chlorine in a manner that is hazardous to human health. Therefore, removing TA from water media is essential. Many methods, such as adsorption, membrane separation, biological processes, ion exchange, coagulation, and chemical oxidation, have been used to remove TA from aqueous solutions [15]. Adsorption is considered the most efficient method because it is less expensive, highly efficient, and straightforward [16].

Because it contains Si–OH and Si–O groups, the exterior of SBA-15 has a negative charge density. This makes it unsuitable for TA adsorption because organic molecules have a negative charge. Surface modification of SBA-15 with amino groups is effective in improving the adsorption activity. Castaldo et al. [17] prepared amino-functionalized hyper-crosslinked resins through a nitration-reduction process. Functionalization can promote the evolution of microporous structures, which effectively increase the adsorption of polar dyes and CO_2_. Melnyk et al. [18] used a one-stage sol-gel method to synthesize amino silica spherical particles. The highest adsorption capacity values for acid red, fluorescein, and methylene blue dyes were 262, 132, and 146 mg/g, respectively. Tang et al. [19] synthesized polyethyleneimine-modified silica nanoparticles containing abundant amino groups. The maximum adsorption capability of acid organic II was as high as 705.3 mg/g. Babapour et al. [20] used a solvothermal method to fabricate a metal organic framework (MOF), which was then amino functionalized using triethylamine. The highest adsorption amount for Cr (VI) was 78.12 mg/g. Huang et al. [21] used the functionalization of chitosan by using tetraethylenepentamine to adsorb eosin Y dye. The highest amount of adsorption (292.4 mg/g) was observed at 25 °C.

In this study, two types of amino groups (aminopropyltriethoxysilane and pentaethylene hexamine) were used for the functionalization of SBA-15 to improve TA adsorption. SBA-15 was prepared with a surfactant template and sodium silicate precursor by using a hydrothermal treatment method. The effects of the calcination temperature, calcination time, hydrothermal treatment temperature, and hydrothermal treatment time on the pore textures of the SBA-15 specimens were observed. The surface characteristics and physical properties of specimens were examined. Furthermore, the adsorption of TA on SBA-15 materials after surface modification of amino groups was explored. The influences of the experimental parameters, such as solution temperature, solution pH, agitation speed, adsorbent dosage, and initial TA concentration, on the elimination of TA were also explored. Finally, the thermodynamics (entropy, enthalpy, and Gibbs free energy), isotherm adsorption (Freundlich and Langmuir equations), and kinetics data (pseudo-first-order and pseudo-second-order models) were evaluated.

## 2. Materials and Methods

### 2.1. Materials

Sodium silicate (Na_2_O(SiO_2_)_x_(H_2_O)_x_, SiO_2_ ~26.5 wt.%, Na_2_O ~10.6 wt.%), 3-aminopropyltriethoxysilane (APTES), pluronic triblock copolymer (P123, EO_20_PO_70_EO_20_), and TA (C_76_H_52_O_46_, 1701 g/mol) were obtained from Sigma-Aldrich (Taufkirchen, Germany). Pentaethylene hexamine (PEHA), ethanol (99.9 wt.%), hydrochloric acid, sodium hydroxide, and sulfuric acid were purchased from Merck (Gernsheim, Germany). High-purity (99.995%) mixed gas (79% N_2_ and 21% O_2_) was purchased from Sun Fu (Taipei, Taiwan).

### 2.2. Synthesis of the SBA-15 Materials

The SBA-15 specimens were fabricated utilizing sodium silicate as the silicon precursor and P123 as the template [22,23]. For a representative experiment, 2.0 g of P123 was added to 60 mL of 2.0 M HCl solution under continuous stirring at 300 rpm and 35 °C. Then, sodium silicate (5.2 g) was dissolved slowly into the P123 solution with agitation at 600 rpm and 35 °C. The suspension was stirred for 24 h, and then placed in a Teflon-lined autoclave. After heating the mixture at 100 °C for 24 h, a white precipitate was collected through filtration and washed by distilled water. The solid was dried at 60 °C for 24 h. Then, SBA-15 was acquired by calcining the solid in air at 550 °C for 6 h.

### 2.3. Synthesis of the Amino-Functionalized SBA-15 Specimens

**Figure 1:**
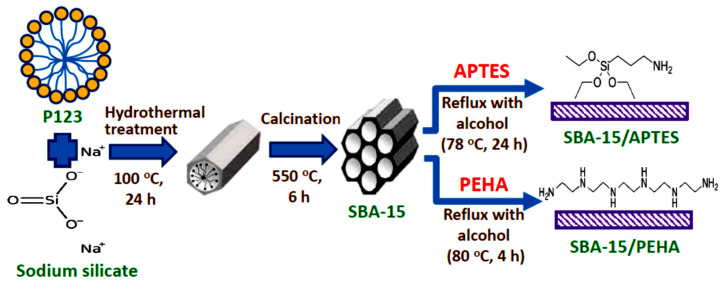
Schematic representation of SBA-15, SBA-15/APTES, and SBA-15/PEHA synthesis.

Using a reflux apparatus, 2.0 g of SBA-15 and 2.0 g of APTES were mixed in 60 mL of anhydrous alcohol at 78 °C for 24 h [24]. The sediment was repeatedly rinsed with ethanol, and then dried in a vacuum oven at 60 °C. The collected sample was marked as SBA-15/APTES. In addition, 1.0 g of PEHA was dissolved in 50 mL of ethanol under agitation for 40 min [25]. After adding 2.0 g of SBA-15, the mixture was heated under reflux at 80 °C for 4 h. Finally, the specimen was dried in a vacuum oven at 60 °C. The collected specimen was marked as SBA-15/PEHA. Figure 1 shows a schematic representation for synthesis of amino-functionalized SBA-15 specimens.

### 2.4. Characterization of the Silica Specimens

The pore characteristics and surface area of the mesoporous silica specimens were investigated using a N_2_ sorption experiment at 77 K by using a Micrometrics ASAP 2020 apparatus (Norcross, GA, USA). The surface functional groups of the amino-modified SBA-15 samples were inspected by a Shimadzu FTIR-8300 analyzer (Nakagyo-ku, Kyoto, Japan). The mesophases and crystalline phases of the silica specimens were assembled on an X’pert pro system X-ray diffractometer (XRD, PANalytical, Malvern, UK) equipped with Cu-kα radiation. The mesoporous structure of the functionalized and unfunctionalized silica materials was inspected by a JEM-2100 (JEOL, Akishima, Tokyo, Japan) transmission electron microscope (TEM). The surface morphologies of the silica specimens were observed through an S-3400N (HITCHI, Chiyoda, Tokyo, Japan) field-emission scanning electron microscope (SEM). The thermal decomposition path of the uncalcined SBA-15 was observed through a thermogravimetric analyzer (TGA, Mettler Toledo, OH, USA, model TGA/SDTA851e). W/W_0_ represented the remaining mass of the sample.

### 2.5. Adsorption Studies

The adsorption experiments were performed to observe the adsorption performance of the mesoporous silica materials by using a bath method to study changes in solution temperature, solution pH, agitation speed, initial TA concentration, and adsorbent dosage. For a typical experiment, 50 mg of adsorbent (SBA-15, SBA-15/APTES, or SBA-15/PEHA) was put into 250 mL of the TA solution, and the solution was agitated constantly. The initial TA concentrations were changed from 10 to 50 mg/L, adsorbent dosages from 10 to 100 mg, agitation speed from 100 to 250 rpm, solution pH from 2 to 5, and solution temperatures from 25 to 60 °C. After adsorption, the residual concentrations of TA at constant periods from 0 to 120 min were determined using the ultraviolet–visible Genesys spectrophotometer (Thermo Electron Corporation, Waltham, MA, USA). The maximum absorbance wavelength was 278 nm. A standard solution was used to calibrate the UV–visible spectrophotometer for every analysis. The amount of TA adsorption capacity (*q_t_*, mg/g) and removal efficiency (*R*, %) of the adsorbents were evaluated using the formulas:

(1) $$q_{t}=\frac{(C_{0}-C_{t})V}{W}$$

(2) $$R=\frac{C_{0}-C_{t}}{C_{0}}\times 100$$

where *W* (g) is the mass of adsorbent, *C*_0_ and *C_t_* (mg/L) are the TA concentrations at the beginning and at time *t*, and *V* (L) is the volume of the TA solution.

## 3. Results and Discussion

### 3.1. Analysis of Phase Features and Surface Functional Groups

**Figure 2:**
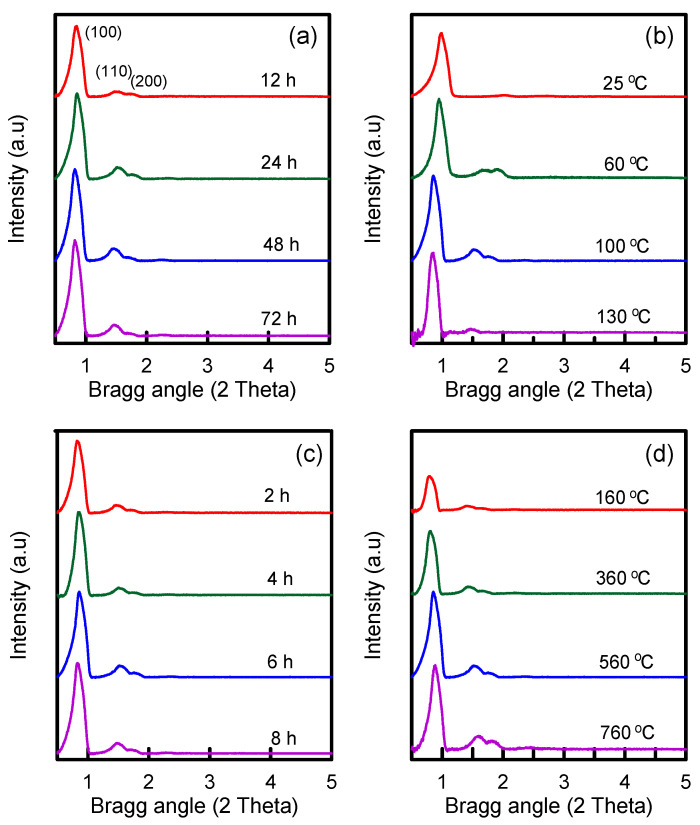
XRD images of SBA-15 samples prepared under various synthesis conditions: (**a**) Hydrothermal treatment time; (**b**) hydrothermal treatment temperature; (**c**) calcination time; (**d**) calcination temperature.

The XRD technique is frequently developed to analyze the characteristics of phase composition of silica materials in synthesis process. Figure 2 presents the XRD patterns with small-angle diffraction of silica samples obtained under different synthesis conditions. These samples exhibited three peaks at (100), (110), and (200) planes, corresponding to two-dimensional and hexagonal SBA-15 mesostructures [26]. Figure 2a displays the XRD images of the SBA-15 prepared at hydrothermal treatment time of 12–72 h. The peak intensity increased with an increase in hydrothermal time, indicating that increasing the hydrothermal time can help the formation of a well-ordered SBA-15 structure. The change in hydrothermal temperature from 25 to 130 °C (Figure 2b) exhibited the same trend as that of the change in hydrothermal time, indicating that enhancing hydrothermal temperature favors the formation of well-ordered mesoporous material. However, when the temperature was enhanced to 130 °C, the (110) and (200) peaks disappeared, indicating that violent hydrothermal reaction may destroy the micellar template, leading to a collapse of pore structure. Therefore, the optimum hydrothermal treatment temperature is 100 °C. The XRD patterns of the silica specimens synthesized at calcination time of 2–8 h are displayed in Figure 2c. The aim of calcination is to eliminate organic surfactants to obtain a porous solid. A high-ordered SBA-15 structure was observed by increasing the calcination time. The optimum calcination time was identified as 6 h. The XRD images of the silica specimens synthesized at calcination temperature of 160–760 °C are presented in Figure 2d. The peak intensity was increased with an increase in calcination temperature, suggesting that increasing the calcination temperature was useful in the synthesis of well-ordered SBA-15 materials with hexagonal symmetry. However, at a low calcination temperature of 160 °C, the three peak intensities were obviously weakened. This is probably because the surfactant template was not removed completely.

**Figure 3:**
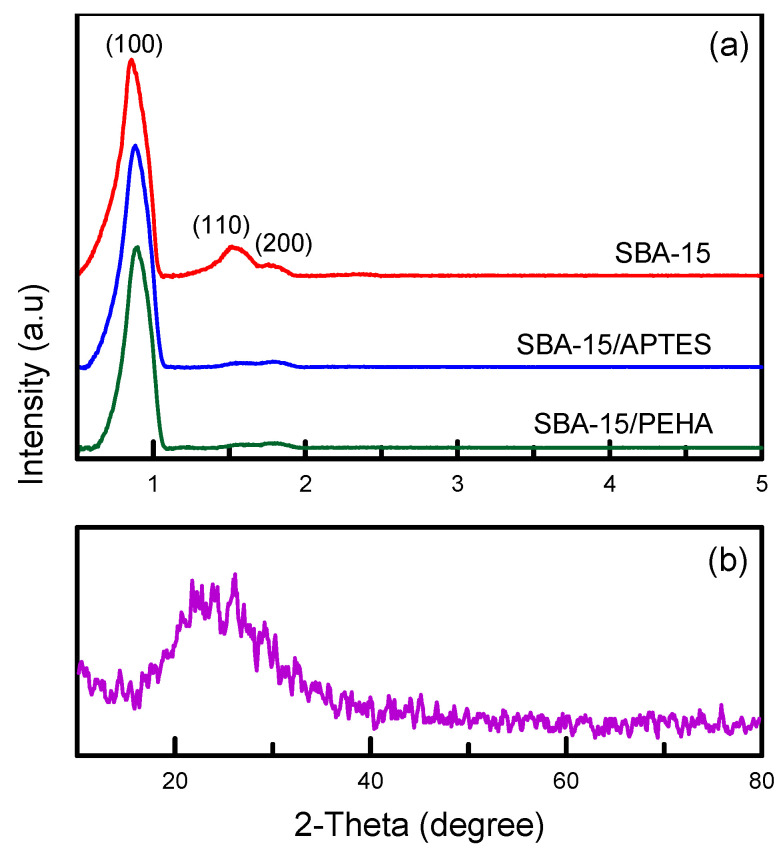
(**a**) XRD images of unfunctionalized and amino-functionalized silica samples; (**b**) SBA-15 sample.

Figure 3a displays XRD images before and after amino-functionalized SBA-15 materials. The XRD data exhibit three peaks at (200), (110), and (100), implying that the pure SBA-15 sample has hexagonal symmetry. In the same situation, the amino-modified samples (SBA-15/APTES and SBA-15/PEHA) also maintained an intense (100) peak and weaker (110) and (200) peaks. The observation further confirmed that both SBA-15/APTES and SBA-15/PEHA maintained a hexagonally ordered structure after functionalization [27]. Figure 3b presents an XRD pattern with a wide-range scan (2Θ = 10°–80°) of the SBA-15 sample. A broad peak presented at diffraction angles from 20° to 30°, indicating the formation of disordered cristobalite [28].

**Figure 4:**
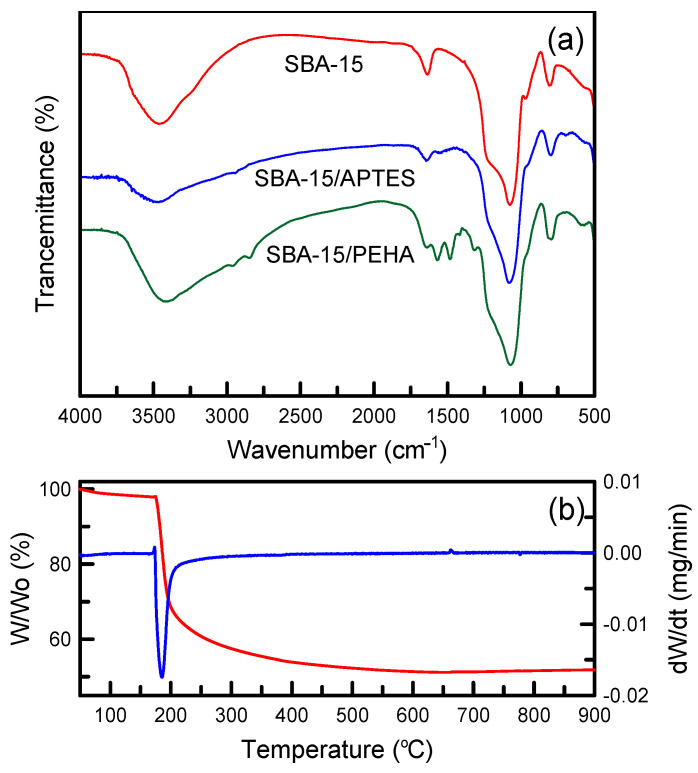
(**a**) FTIR spectra of unfunctionalized and amino-functionalized silica samples; (**b**) TG and DTG thermograms of uncalcined SBA-15 samples.

Qualitative identification of functional groups was accomplished by FTIR spectroscopy. The FTIR spectra of the amino-functionalized and unfunctionalized SBA-15 materials are presented in Figure 4a. In the pure SBA-15 sample, a wide peak at 3200–3700 cm^−1^ was caused by the presence of O–H groups [29]. The C=O vibrations were observed at a band of 1635 cm^−1^. The C=C groups were identified at 1605 cm^−1^, and the band at 1375 cm^−1^ was the –CH_3_ stretching of the methyl group. The band at 980 cm^−1^ represented the appearance of the Si–OH groups, and Si–O–Si groups were located at 790 and 450 cm^−1^ [30]. In the SBA-15/APTES and SBA-15/PEHA samples, amino group stretching vibrations were found at 692, 1499, and 1572 cm^−1^ [31]. This verified that amino groups were grafted onto the silica surface. Anbia and Salehi [32] observed that broad −NH_2_ vibrations appeared at 3250–3450 cm^−1^ in amino-modified silica samples. Nevertheless, the functional group was not easily examined due to the fact that the −NH_2_ bands may be covered by the wide band of the –OH groups. Furthermore, SBA-15/PEHA exhibited a stronger relative amino peak intensity than SBA-15/APTES. Figure 4b displays the TG curve of the uncalcined SBA-15 sample. The mass loss occurring at temperatures lower than 200 °C was caused by water evaporation. The mass loss decreased slowly with an increase in thermal decomposition temperature because a longer heating time was required for the release of the surfactant matter from the high-porosity SBA-15 structure. The differential TG curve revealed only one peak at 195 °C. Similar results were observed by Liou [33] for the thermal decomposition of an uncalcined MCM-41 sample in air.

### 3.2. Analysis of the Pore Structure

**Figure 5:**
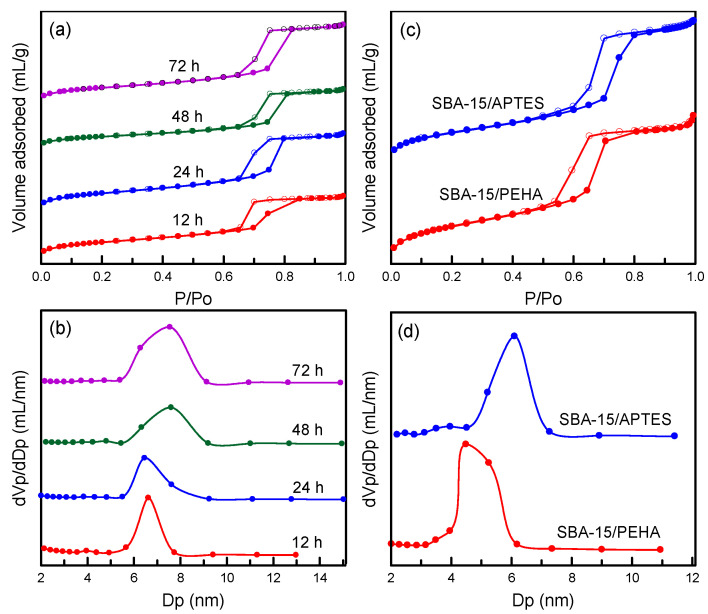
Nitrogen sorption isotherms and pore size distributions: (**a**,**b**) SBA-15 samples at different hydrothermal treatment times; (**c**,**d**) SBA-15/APTES and SBA-15/PEHA samples.

The pore structure is an important physical property of mesoporous materials. Nitrogen isotherm method can standardly determine the porous structure and textural parameters. Figure 5a displays the nitrogen sorption isotherms of the SBA-15 specimens prepared at hydrothermal treatment times of 12–72 h. Pure SBA-15 exhibited a type-IV hysteresis loop with type H1 at a relative pressure of *P/P_o_* = 0.6–0.9, which is characteristic of a mesoporous structure [34]. By comparing the SBA-15 samples prepared at various hydrothermal treatment times, it was revealed that the isotherms were similar. This observation indicates that an increase in hydrothermal time does not affect the mesostructure of the SBA-15 framework. Figure 5b presents the features of pore size distribution. The SBA-15 samples had pore sizes of 6.62–7.60 nm. The SBA-15 obtained at hydrothermal times of 12–24 h had a smaller pore diameter than those obtained at 48–72 h. These samples displayed a narrow and uniform pore distribution, implying that the pore structure of SBA-15 was stable.

The isotherms of the amino-functionalized SBA-15/APTES and SBA-15/PEHA specimens are presented in Figure 5c. The two isotherms also exhibited a type-IV hysteresis loop, implying a mesoporous material. The amino-functionalized SBA-15 samples (Figure 5c) exhibited similar loops to those of pure SBA-15 (Figure 5a), indicating that the combination of SBA-15 and amino groups did not destroy the silica mesostructure. The pore size of SBA-15/APTES (6.10 nm) was larger than that of SBA-15/PEHA (4.49 nm; Figure 5d). Table 1 lists the pore volume, surface area, and mesopore fraction of the unfunctionalized and amino-functionalized silica specimens. The surface areas of the pure SBA-15 samples ranged from 546 to 766 m^2^/g, which decreased with an increase in hydrothermal treatment time. The SBA-15 at a hydrothermal time of 48 h had the largest pore volume (1.105 cm^3^/g). The SBA-15 samples exhibited higher surface area and pore volume as compared with the SBA-15/APTES and SBA-15/PEHA samples. The addition of amino functional groups into silica pores led to reductions in textural parameters. This is consistent with the literature [35]. Moreover, the pore volume and surface area of SBA-15/APTES (0.521 cm^3^/g and 264 m^2^/g) were higher than those of SBA-15/PEHA (0.272 cm^3^/g and 167 m^2^/g). The mesopore fractions of the pure SBA-15 samples ranged from 97.69% to 99.42%, which increased with an increase in hydrothermal time.

### 3.3. Surface Morphology of the Mesoporous Silica Samples

**Figure 6:**
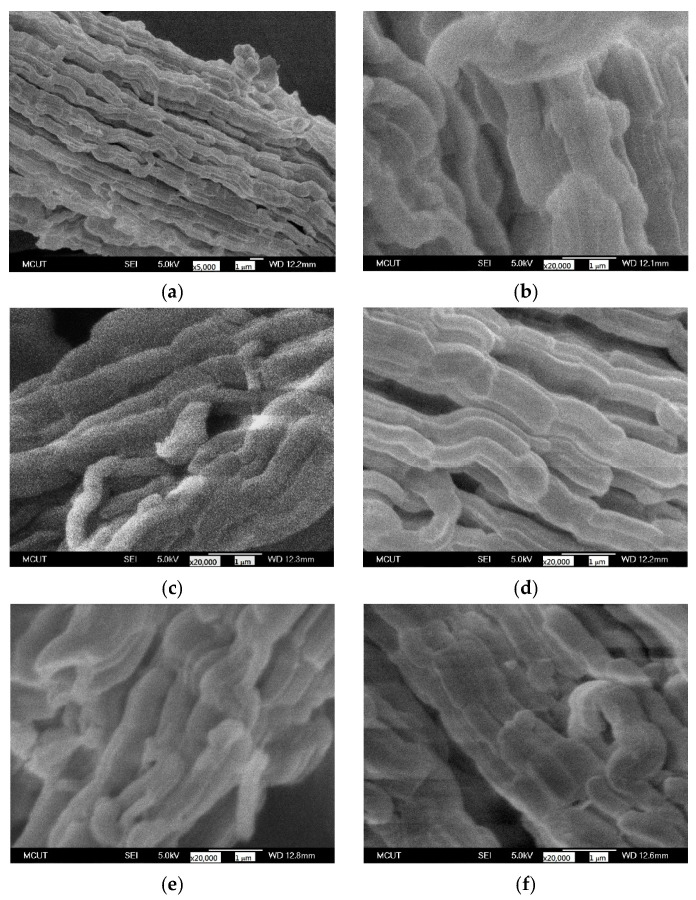
SEM images: (**a**) SBA-15; (**b**–**d**) SBA-15 synthesized at hydrothermal treatment times of 12, 24, and 72 h, respectively; (**e**) SBA-15/APTES; (**f**) SBA-15/PEHA.

The SEM technique was utilized to observe the changes of surface morphology before and after amination of samples. The SEM pictures of SBA-15 without amination are presented in Figure 6a–d. The SBA-15 sample comprised agglomerates of rod-like particles (Figure 6a), consistent with the literature [36], whereas the SBA-15 sample acquired at a hydrothermal treatment time of 12 h exhibited irregular-shaped particles, indicating that the silica structure was not completely developed (Figure 6b). The SBA-15 samples obtained at hydrothermal times of 24 and 72 h had a glossy and regular surface (Figure 6c,d), indicating the formation of organized structures. This is consistent with the XRD measurements in Figure 2a. The width of the SBA-15 particles was in the range between 380 and 830 nm. The morphology of SBA-15/APTES and SBA-15/PEHA (Figure 6e,f) did not vary as much as that of pure SBA-15, but the average width was reduced by adding the amino groups (330–610 nm for SBA-15/APTES and 300–560 nm for SBA-15/PEHA).

**Figure 7:**
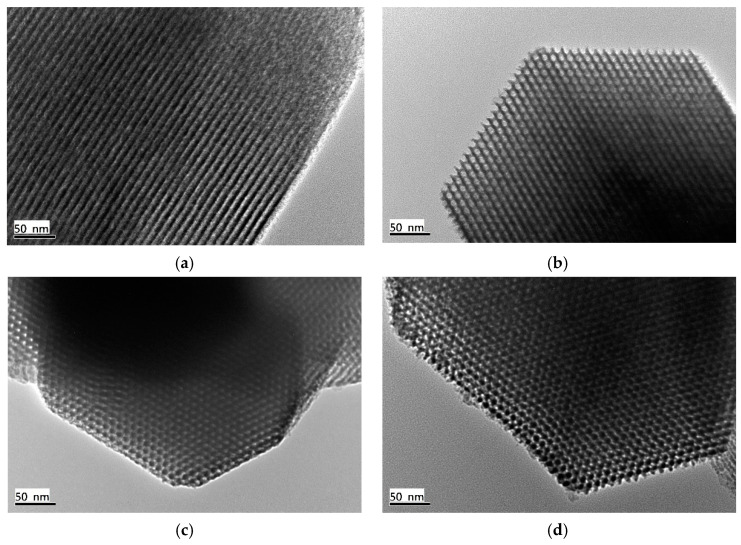
TEM images: (**a**,**b**) SBA-15; (**c**) SBA-15/APTES; (**d**) SBA-15/PEHA.

The TEM characterization technique was utilized to identify the difference of the microstructure of the prepared silica materials. The mesostructures of the amino-functionalized and unfunctionalized SBA-15 samples were investigated using TEM. Figure 7a,b verify that SBA-15 consists of parallel pore channels and highly ordered mesopores. The silica sample also possessed a large-scale hexagonal structure (Figure 7b). The mesostructure was formed through the self-assembly of surfactant micelles and silicate species [37]. The average pore size of 7.80 nm was similar to the N_2_ adsorption test in Figure 5b. After the amination reaction, the SBA-15/APTES (Figure 7c) and SBA-15/PEHA (Figure 7d) morphology was analogous to pure SBA-15. However, the pore diameters were reduced to 6.25 and 5.00 nm, which was attributed to the amino group coating on the silica surface.

### 3.4. TA Adsorption Study

**Figure 8:**
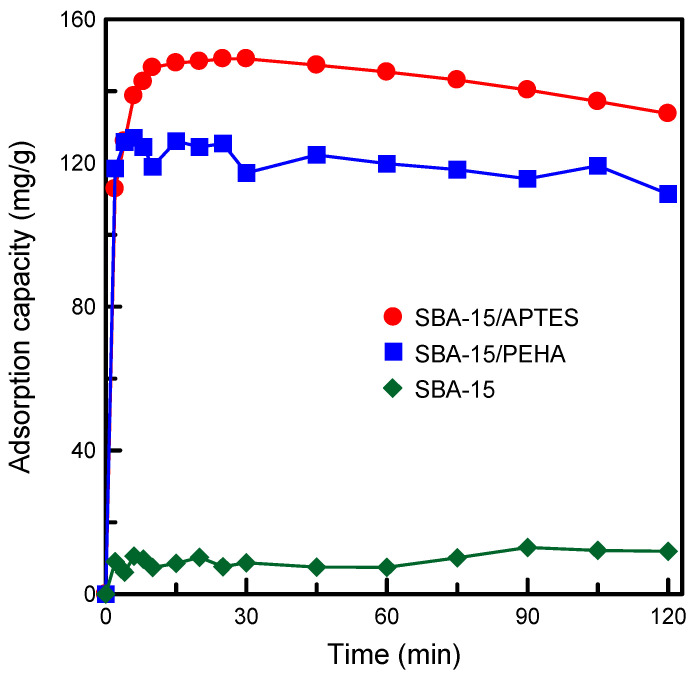
Effects of contact time on the adsorption performance of various kinds of adsorbents.

TA organic compound was utilized as the adsorbate to characterize the adsorption performance of the amino-functionalized SBA-15 materials. In order to evaluate the efficacy of the prepared adsorbents, the equilibrium adsorption of the TA was studied by changing various operating conditions. In the study, different types of amino-modified silica samples were used to remove TA from the aqueous solution. The influence of contact time on the adsorption capacities of adsorbents is exhibited in Figure 8. The adsorption capacity decreased in the following order: SBA-15/APTES > SBA-15/PEHA > pure SBA-15. The hydroxyl groups on the silica surface could not supply adsorption sites sufficiently strong to interact with the TA molecules. Therefore, the pure SBA-15 sample had the lowest adsorption performance. The amino-modified SBA-15 specimens showing the higher adsorption capacity can be explained as follows: Typically, amino groups are favorable to the adsorption of anionic species, and TA is an anionic compound. In the aqueous solution, the electrostatic attraction between the negatively charged TA molecules and positively charged amino groups on the SBA-15 surface (−NH_3_^+^) led to an increase in TA adsorption [32]. Furthermore, the SBA-15/APTES specimen had a higher surface area and pore volume than the SBA-15/PEHA specimen (Table 1) and, consequently, SBA-15/APTES exhibited more efficient adsorption than SBA-15/PEHA. The contact time curves also demonstrated that TA uptake was rapid in the first few minutes, and then proceeded at a slower rate. The rapid adsorption during the initial adsorption period was because the adsorbent surface was unoccupied. After the adsorption reached the saturation point, the slow permeation of TA into the silica pores resulted in a reduction in the adsorption rate.

**Figure 9:**
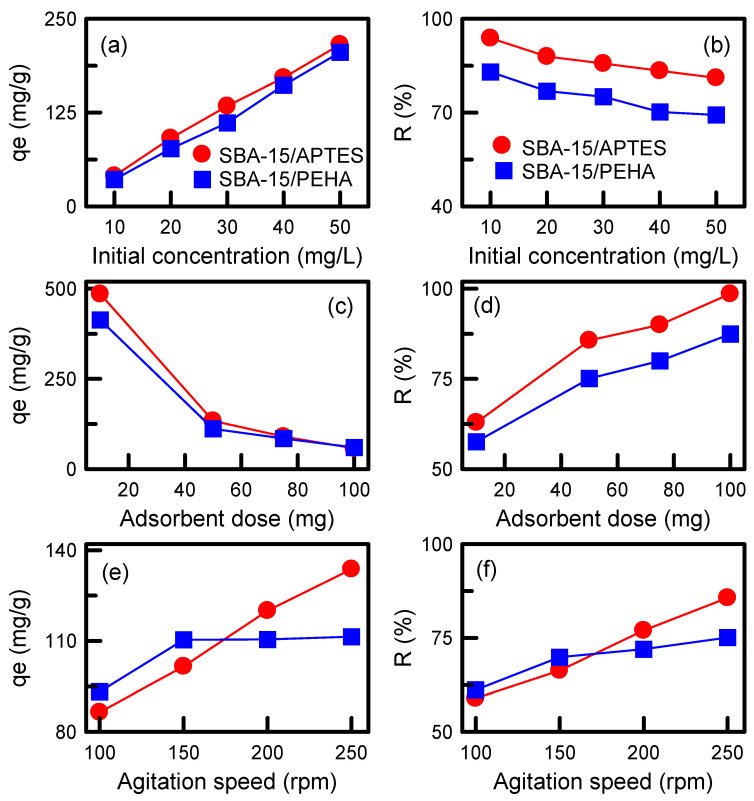
Effects of various adsorption conditions on the adsorption capacity and removal efficiency of adsorbents: (**a**,**b**) Initial TA concentration; (**c**,**d**) adsorbent dosage; (**e**,**f**) agitation speed.

Figure 9a,b illustrates the influence of the initial TA concentration on adsorption capacity and the removal efficiency of amino-modified adsorbents. The adsorption capacity increased rapidly with an increase in the initial TA concentration. A higher initial TA concentration resulted in an increased mass gradient between the solute and adsorbent, which accelerated the diffusion of the TA molecules onto the SBA-15 surface. In addition, collisions between the TA and adsorbent also increased when the initial TA concentration increased. These results enhance the adsorption activity. By contrast, the removal efficiency decreased with an increase in the initial TA concentration. This was also observed in the elimination of reactive blue 21 dye from manganese oxide nanoparticles [38]. The SBA-15/APTES samples revealed a higher adsorption capacity and removal efficiency as compared with those of SBA-15/PEHA. For SBA-15/APTES specimen, a maximum removal efficiency of approximately 94% was observed at an initial concentration of 10 mg/L, suggesting that the adsorbent had sufficient ability to treat trace pollutants.

The effect of adsorbent dosage on the adsorption capacity and removal efficiency of the adsorbents is displayed in Figure 9c,d. The results indicate that increasing the adsorbent dosage from 10 to 100 mg increases the removal efficiency but decreases the adsorption capacity. Removal efficiency is increased because the available surface areas and adsorption sites are increased, whereas the reduction in adsorption capacity is because the adsorption sites are not saturated. This is consistent with a report by El-Sewify et al. on the adsorption of malachite green dye on zinc MOFs [39]. The SBA-15/APTES and SBA-15/PEHA samples displayed the maximum adsorption capacities of 485.15 and 423.33 mg/g and the highest removal efficiency of 98.61% and 87.43%, respectively.

Agitation speed can influence the dispersion of solute in the solution, which is an important factor in the adsorption process. The influence of agitation speed on adsorption capacity and removal efficiency is illustrated in Figure 9e,f. For the SBA-15/APTES specimen, the removal efficiency and adsorption capacity were enhanced with an increase in the agitation speed, whereas the adsorption capacity and removal efficiency were enhanced for the SBA-15/PEHA specimen when the agitation speed increased from 100 to 150 rpm, and then remained constant. Increasing the agitation speed can reduce the film resistance of the adsorbent, resulting in an increase in TA adsorption.

**Figure 10:**
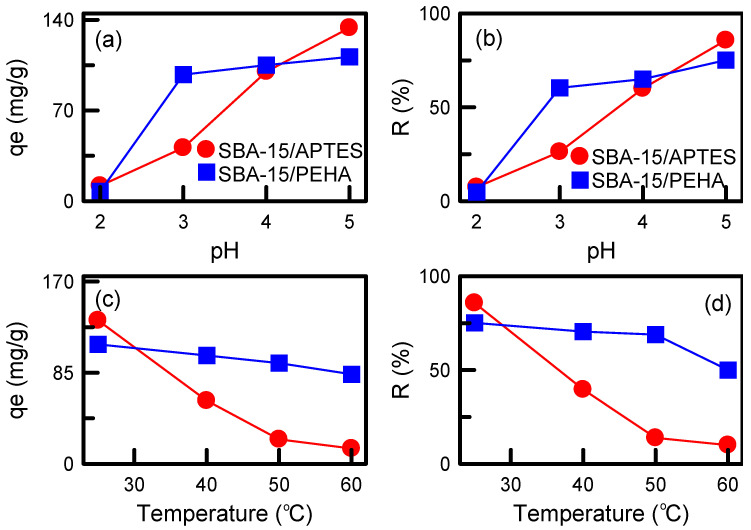
Effects of various adsorption conditions on the adsorption capacity and removal efficiency of adsorbents: (**a**,**b**) Solution pH; (**c**,**d**) solution temperature.

The influence of solution pH on the adsorption capacity and removal efficiency of the adsorbents is presented in Figure 10a,b. The removal efficiency and adsorption capacity were increased with an increase in the pH values. The maximum adsorption capacities of the two adsorbents (133.76 and 111.37 mg/g) were observed at pH 5. TA exists in its molecular form at pH 4.5. The adsorption capacity at pH 5 was enhanced because of the increasing hydrogen bonding and electrostatic force attraction between the TA molecules and SBA-15 surface [31]. However, TA is an anionic molecule, and at a pH of 8–11, the negatively charged sites on the adsorbent surface increased. The existence of excess OH^−^ ions compete with the TA anions, and result in the dissociation of TA during the adsorption process. Hence, this study focused on TA adsorption in acidic media.

The influence of solution temperature on the adsorption of TA is presented in Figure 10c,d. The adsorption capacity and removal efficiency were increased with a decrease in the solution temperature. The maximum removal efficiency and adsorption capacity presented at 25 °C, indicating an exothermic process. Temperature was a key factor for SBA-15/APTES in the adsorption process. However, SBA-15/PEHA was not sensitive to the solution temperature. Increasing solution temperature may cause an increase in TA solubility, weakening the interaction forces between the adsorbent and adsorbate and decreasing TA adsorption. Koyuncu and Okur [40] observed similar behavior in the adsorption of acid violet 90 dye on ordered mesoporous carbon.

### 3.5. Thermodynamic Studies

Thermodynamic parameters provide in-depth information on internal energy changes associated with adsorption. The standard free energies (Δ*G*, kJ/mol) for the adsorption of TA onto SBA-15/APTES and SBA-15/PEHA were calculated using the thermodynamic equilibrium constant (*K_c_*), which is written as below [41]:

Δ*G* = −*RT* ln*K_c_* (3)

(4) $$K_{c}=\frac{C_{s}}{C_{e}}$$

where *R* is the gas constant (8.314 J/mol-K); *T* is the absolute temperature (*K*); and *C_s_* and *C_e_* (mg/L) are the equilibrium concentration of TA on the solid and in the solution, respectively.

The enthalpy (Δ*H*, kJ/mol) and entropy (Δ*S*, J/mol-K) can be estimated by the van’t Hoff equation:

(5) $$\ln K_{c}=\frac{\Delta S}{R}-\frac{\Delta H}{RT}$$

**Figure 11:**
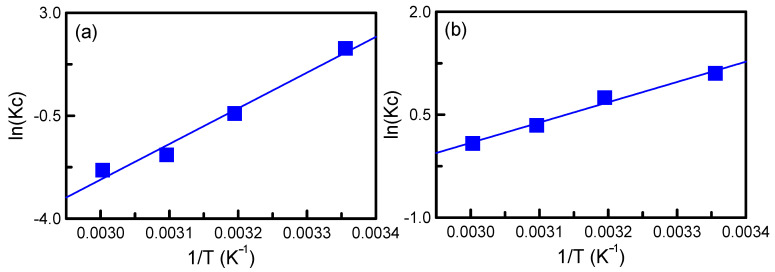
Plot of equilibrium constant versus temperature by using Van’t Hoff equation: (**a**) SBA-15/APTES; (**b**) SBA-15/PEHA.

Δ*S* and Δ*H* can be acquired from the intercept and slope of Equation (5) (Figure 11), and Table 2 lists the thermodynamic parameters. The Δ*G* values were negative at 25 and 40 °C, indicating that adsorption was spontaneous. However, the Δ*G* values increased with an increase in temperature, suggesting that the adsorption process was not feasible at a high temperature. The negative values of Δ*H* (−100.99 and −24.58 kJ/mol) further confirmed that the adsorption process was exothermic. The negative values of Δ*S* (−324.99 and −72.99 J/mol-K) reflect the decrease in the freedom of mobility of TA at the liquid–solid interface of the adsorption process [42].

### 3.6. Adsorption Isotherm Experiments

The adsorption equilibrium between the solid and liquid surfaces in the adsorption processes can be characterized using the Langmuir and Freundlich equations. The adsorption isotherm experiments were performed using 10 mg of SBA-15/APTES or SBA-15/PEHA in 50 mL of TA solution. To reach adsorption equilibrium, the solution was stirred for 24 h. The concentrations of TA were altered from 10 to 50 mg/L. The solution pH was 5.

The linear forms of the Langmuir and Freundlich equations are [43]:

(6) $$\frac{1}{q_{e}}=\frac{1}{q_{L}}+\frac{1}{q_{L}K_{L}C_{e}}$$

(7) $$\log q_{e}=\log K_{F}+\frac{1}{n}\log C_{e}$$

where *C_e_* (mg/L) is the equilibrium concentration, *q_e_* (mg/g) is the adsorption capacity, *K*_F_ and *n* are the Freundlich constants, *K_L_* (mL/mg) is the Langmuir constant, and *q_L_* (mg/g) is the maximum adsorption capacity.

**Figure 12:**
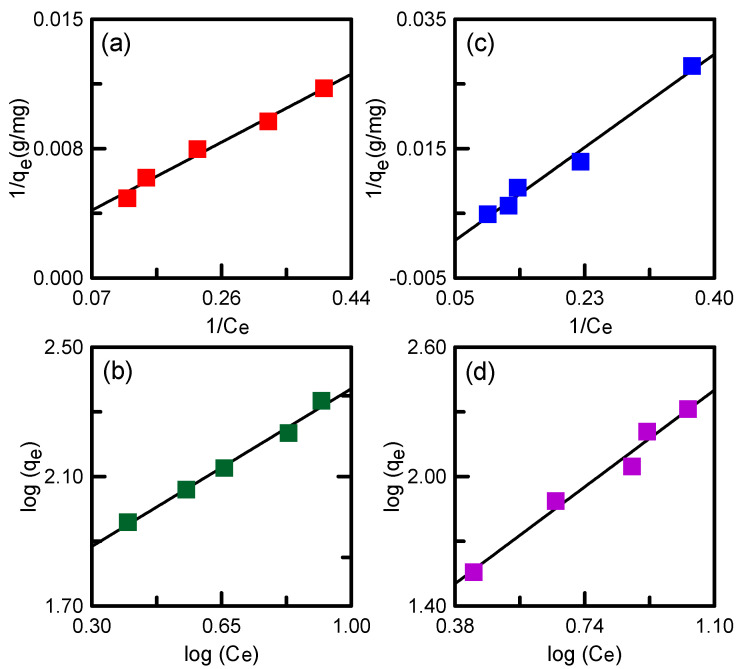
Plots of Langmuir model for (**a**) SBA-15/APTES and (**c**) SBA-15/PEHA; Freundlich model for (**b**) SBA-15/APTES and (**d**) SBA-15/PEHA.

The Freundlich and Langmuir equation plots are displayed in Figure 12, and the corresponding parameters are given in Table 3. The *R*^2^ value was employed to estimate the fit of the models to the experimental data. The adsorption of TA onto SBA-15/APTES and SBA-15/PEHA followed both Langmuir and Freundlich isotherms, as shown by *R*^2^ values (0.98–0.99) in Table 3 [44]. The maximum adsorption capacities (*q_L_*) of SBA-15/APTES and SBA-15/PEHA were 418.41 and 303.03 mg/g, respectively. The R_L_ values of the two adsorbents were within the range of 0–1, with *n* > 1 indicating an advantageous adsorption course. This result implies that adsorption was likely to occur [45].

### 3.7. Kinetic Studies

To further realize the adsorption mechanism, pseudo-first-order and pseudo-second-order models were examined. The equations for the two models are [46]:

(8) $$q_{t}=q_{e}(1-e^{-k_{1}t})$$

(9) $$\frac{t}{q_{t}}=\frac{1}{k_{2}q_{2}^{2}}+\frac{t}{q_{e}}$$

where *k*_1_ and *k*_2_ are the rate constants of each model, respectively; *q_t_* and *q_e_* (mg/g) are the adsorption capacities of adsorbents at time *t* and at equilibrium, respectively. The solution pH was 5.

**Figure 13:**
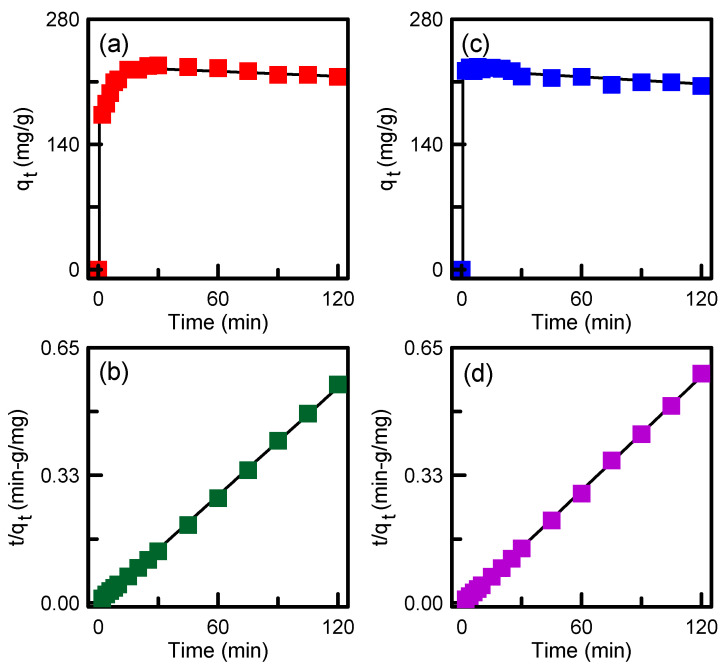
Plots of pseudo-first-order model for (**a**) SBA-15/APTES and (**c**) SBA-15/PEHA; pseudo-second-order model for (**b**) SBA-15/APTES and (**d**) SBA-15/PEHA.

Adsorption capacity versus time using Equations (8) and (9) is plotted in Figure 13. In Table 4, *R*^2^ indicates that the pseudo-second-order model provides the optimum fitting for the adsorption of TA onto SBA-15/APTES and SBA-15/PEHA, suggesting that the adsorption was mainly controlled by chemisorption. Furthermore, the experimental *q_e_* values (215.78 and 205.52 mg/g) are similar to the calculated *q_e_* values (218.34 and 206.61 mg/g).

Table 5 displays the maximum adsorption capacity of TA onto different adsorbents. The SBA-15/APTES and SBA-15/PEHA samples prepared in this study had high adsorption capacities (485 and 413 mg/g, respectively) as compared with adsorbents used in other studies. Thus, the amino-functionalized and mesoporous silica-based materials synthesized in this work could be utilized as efficient adsorbents for the elimination of TA from aqueous solutions.

## 4. Conclusions

The preparation of amino-functionalized SBA-15 composites for the adsorption of TA was investigated in the present work. The XRD observations indicate that the mesostructure of SBA-15 materials is highly sensitive to hydrothermal time, hydrothermal temperature, calcination time, and calcination temperature. The FTIR analysis revealed that amino groups were successfully grafted onto the SBA-15 surface, and TEM verified that the silica pore framework remained nearly unchanged after the amination reaction. The surface area and pore volume of the adsorbents were SBA-15 > SBA-15/APTES > SBA-15/PEHA. The adsorption efficiency of SBA-15 material was enhanced by way of surface modification with amino groups, and the adsorption capacity increased because of the formation of an electrostatic interaction and hydrogen bond between the adsorbent surface and TA molecules. SBA-15/APTES exhibited higher adsorption activity for the removal of TA than SBA-15/PEHA. The adsorption capacities were increased with increases in the initial TA concentration, agitation speed, and solution pH. However, increasing the adsorbent dosage and solution temperature resulted in a reduction in adsorption capacity. Pure SBA-15 had little affinity for TA in aqueous solution. Amino-functionalized SBA-15 presented higher TA adsorption efficiency than pure SBA-15. The results of the study identified high-efficiency adsorbents that could be applied to the removal of organic pollutants. As future perspectives, we propose to use magnetic iron compounds to facilitate the separation for larger scale application, as well as to adsorb other pollutants of interest, for example, hazardous heavy metals, and finally to a real industrial wastewater.

## Figures and Tables

**Table 1:** Surface area, pore volume, and pore diameter of silica specimens.

Sample	S_BET_ (m^2^/g)	*V_t_* (cm^3^/g)	*V_mic_* (cm^3^/g)	*V_meso_* (cm^3^/g)	*V_meso_/V_t_* (%)	*d_p_* (nm)
SBA-15–12 h	766	1.038	0.024	1.014	97.69	6.62
SBA-15–24 h	655	1.044	0.014	1.030	98.66	6.46
SBA-15–48 h	604	1.105	0.009	1.096	99.19	7.60
SBA-15–72 h	546	1.037	0.006	1.031	99.42	7.55
SBA-15/APTES	264	0.521	0.005	0.516	99.04	6.10
SBA-15/PEHA	167	0.272	0.005	0.267	98.16	4.49

S_BET_, specific surface area; *V_t_*, total pore volume; *V_mic_*, micropore volume; *V_meso_*, mesopore volume; *d_p_*, pore diameter (BJH desorption).

**Table 2:** Thermodynamic parameters for TA adsorption onto amino-modified silica specimens.

Sample	ΔS (J/K × mol)	ΔH (kJ/mol)	ΔG (kJ/mol)			
			25 °C	40 °C	50 °C	60 °C
SBA-15/APTES	−324.99	−100.99	−4.434	−1.096	4.909	6.507
SBA-15/PEHA	−72.99	−24.58	−2.734	−1.953	−0.926	−0.227

**Table 3:** Isotherm constants for TA adsorption onto amino-modified silica specimens.

Sample	Langmuir				Freundlich		
	*R_L_*	*q_L_* (mg/g)	*K_L_*	*R* ^2^	*n*	*K_F_* (mg/g)	*R* ^2^
SBA-15/APTES	0.154	418.41	0.11	0.986131	1.44	47.49	0.990632
SBA-15/PEHA	0.333	303.03	0.04	0.984151	1.02	10.7	0.978013

**Table 4:** Kinetics constants for TA adsorption onto amino-modified silica specimens.

Model	Parameter	Value	
		SBA-15/APTES	SBA-15/PEHA
Pseudo-first-order adsorption kinetic	*q_e,experiment_* (mg/g)	215.78	205.52
	*q_e,calculated_* (mg/g)	219.25	218.08
	*k*_1_ (min^−1^)	0.6473	43.49
	*R* ^2^	0.97224	0.98146
Pseudo-second-order adsorption kinetic	*q_e,experiment_* (mg/g)	215.78	205.52
	*q_e,calculated_* (mg/g)	218.34	206.61
	*k*_2_ (min^−1^)	0.02	0.01
	*R* ^2^	0.99971	0.99978

**Table 5:** Maximum adsorption capacity for TA adsorption onto various adsorbents.

Adsorbent	*q* (mg/g)	Reference
Montmorillonite	219	[47]
Activated carbon	162	[48]
Clay	138	[49]
Polyaniline	117	[50]
Chitosan/NaOH/fly ash composite	244	[51]
Porous crosslinked polystyrene	248	[52]
Biochar	87	[53]
SBA-15/APTES	485	Present work
SBA-15/PEHA	413	Present work

## Data Availability

Data sharing not applicable.
